# Ministers and Physicians

**Published:** 1850-09

**Authors:** 


### ARTICLE V.

### MINISTERS AND PHYSICIANS.

The *Puritan Recorder* is disposed to treat the threatened relation of these two professions with some humor as well as sense. It says:—

"Of late, however, there seem to be symptoms of unhealthy feeling on the part of the medical gentlemen. It is even rumored, that, in some of their Conventions and Societies, they are agitating the project of a breach of the peace with the ministers of the Gespel, because a few of these have soiled their cloth by patronizing and puffing some of the patent nostrums of the day. We have no apology to make for such unministerial conduct, which is highly indiscreet and reprehensible. But this we will say, that, for one respectable and educated clergyman who has stultified himself by being thus mixed up with quackery, at least an hundred have abhorred the contact of 'the unclean thing.'

Why should the whole body of ministers in regular standing, be made responsible for the errors and follies of a few of their brethren, who, in such matters, aie under no ecclesiastical control? Have any of the ecclesiastical bodies, large or small, taken action in the piemises. Has the General Association of Massachusetts recommended to the churches the use of Brandreth's Pills? Has the General Assembly of the Presbyterian Church (Old School), espoused the cause of Sand's Sarsaparilla; or has the New School Assembly taken up the claims of the rival syrup of Townsend? Have the Episcopal bishops given vogue to the big-bellied bottles of Mrs. Kidder's Cordial? Has the General Conference of the Methodist brethren endorsed for the virtues of the Thompsonian hot-drops? Is the American Unitarian Association responsible for Homeopathic doses in medicine as well as in religion? Or has the Baptist communion pledged itself to Hydropathy for any but spiritual purposes? If no such ecclesiastical absurdity has been perpetrated, why is an entire body of unoffending men charged with the extravagances of a few visionary or eccentric brethren, who have ignorantly misapplied their official influence to the suppor t of imposture?

Here we are bound to warn the gentlemen of the medi cal profession, that, if they declare war with the ministers on such grounds, they may yet be obliged to drink out of their own measure. We would inquire whether none of them have been known to patronize quack preachers, empirical reformers, and other religious Jack-puddings, who are vending their noxious nostrums in theology and morals, poisoning and stupifying the minds of the people, and spreading the ravages of spiritual death? Is it not a fact, that very many regular-bred physicians haunt the shops of the most notorious theological impostors, and go abroad to bring their pernicious wares into general use? And now what propriety would there be in preaching up a crusade on the part of the clergy against the physicians, as a class essentially radical and faithless?—*Prairie Herald.*

